# Mexican oregano essential oils as alternatives to butylated hydroxytoluene to improve the shelf life of ground beef

**DOI:** 10.1002/fsn3.1767

**Published:** 2020-07-08

**Authors:** Jesús A. Cantú‐Valdéz, Guadalupe Gutiérrez‐Soto, Carlos A. Hernández‐Martínez, Sugey R. Sinagawa‐García, Armando Quintero‐Ramos, Michael E. Hume, Daniela D. Herrera‐Balandrano, Gerardo Méndez‐Zamora

**Affiliations:** ^1^ General Escobedo Universidad Autónoma de Nuevo León Facultad de Agronomía Nuevo León México; ^2^ Campus Universitario Universidad Autónoma de Chihuahua Facultad de Ciencias Químicas Circuito Universitario s/n Chihuahua México; ^3^ U.S. Department of Agriculture Food and Feed Safety Research Unit Southern Plains Agricultural Research Center Agricultural Research Service College Station TX USA; ^4^ Institute of Agro‐Product Processing Jiangsu Academy of Agricultural Sciences Nanjing China

**Keywords:** antioxidant, essential, microbiology, sensory, texture

## Abstract

Oregano essential oils from *Lippia berlandieri* Schauer (Lb) and *Poliomintha longiflora* Gray (Pl) were tested against the antioxidant butylated hydroxytoluene (Bht) to evaluate effects on the shelf life of ground beef (GB) over 7 days of storage at 4°C. The treatments were GB1 = GB control, GB2 = GB +100 mg/kg of Bht, GB3 = GB +100 mg/kg of Lb, and GB4 = GB +100 mg/kg of Pl. Lightness, redness, hardness, and springiness showed differences (*p* < .05) between treatments and days interaction, which serve as indicators of ground beef preserved quality and consumer acceptance. Mesophilic, psychrophilic, and lactic acid bacteria numbers and antioxidant activity showed differences (*p* < .05) for treatments and days. Sensory attributes showed no differences between treatments. The oregano oils may provide extended shelf life for packaged meat products treated with these natural additives and hence may be used for ground beef preservation.

## 1 INTRODUCTION

Oxidation factors into the quality and acceptability of meat products, affecting attributes such as taste, color, texture, and nutritional value (Ribeiro et al., 2019). Ground beef is such a popular meat food that the beef industry expends major efforts to development strategies to improve shelf life and color stability (Rogers et al., 2014). Warriss (2000) indicated that meat appearance is the principal deciding factor for consumers. Ribeiro et al. (2019) indicated that antioxidants are widely used to prevent oxidation and preserve sensory attributes, antioxidants such as ascorbic acid, butylated hydroxytoluene (Bht), butylated hydroxyanisol (Bha), nitrites, and nitrates. Butylated hydroxytoluene is one of the most commonly used antioxidants and is recognized as safe for use in foods containing fats, pharmaceuticals, petroleum products, rubber, and oil industries, has a low‐molecular weight, is a white crystalline solid, and is a nonstaining hindered‐phenol antioxidant (Yehye et al., 2015). The General Standard for Food Additives CODEX STAN^192^‐, ^1995^ (revision 2019; 08.3 point, Processed comminuted meat, poultry, and game products, considering the notes 15, 130, 162, XS88, XS89, and XS98) on Bht used in foods recommend doses of 100 mg/kg. Ribeiro et al. (2019) stated that the ingestion of Bht and Bha in the body is followed by increases in blood lipid and cholesterol levels, increased synthesis of liver enzymes for Bht metabolism, and is associated with the destruction of compounds such as vitamin D and the onset of urticarial and eczematous dermatitis.

Accordingly, the use of natural antioxidants in meat products potentially offers alternatives to reduce the consumption of synthetic additives (Karre, Lopez, & Getty, 2013). These natural antioxidants, when consumed, exhibit signs of low toxicity and exhibiting functional attributes beneficial to human health controlling and preventing cardiovascular diseases, diabetes mellitus, obesity, and neoplasms (Ribeiro et al., 2019). Several studies have evaluated the preservation of fresh ground meat with plant parts or extracts (Balentine, Crandall, O’Bryan, Duong, & Pohlman, 2006; Biswas, Chatli, & Sahoo, 2012; Dhifi, Jazi, El Beyrouthy, Sadaka, & Mnif, 2020; Kim et al., 2013; Najjaa et al., 2020; Yin & Cheng, 2003), grape seed extract (Gómez, Beriain, Mendizabal, Realini, & Purroy, 2016), mushrooms (*Agaricus bisporus*) (Alnoumani, Ataman, & Were, 2017), synthetic antioxidants or irradiation (Ayari, Han, Vu, & Lacroix, 2016; Lee et al., 2012; Mohamed, Mansour, & Farag, 2011; Yang et al., 2011), and modified atmosphere packaging held under temperature abuse (Lyte et al., 2016). Additionally, natural antioxidants such as aromatic plant oils have been used for meat preservation; for example, antioxidants in oregano, grape seed, cranberry, sage, and thyme decrease oxidation in a variety of products (Corral, Salvador, & Flores, 2013).

Essential oils from several oregano varieties, such as European oregano (*Origanum vulgare* L. and *Origanum onites* sp. A. *sativum* L.) and Mexican oregano (*Lippia berlandieri* Schauer and *Poliomintha longiflora* Gray), have been used to improve the quality of chorizo, breast marinated, and chicken breast quality (Perales‐Jasso *et al*., 2018; Méndez‐Zamora, García Macías, Santellano‐Estrada, Durán Meléndez, & Silva‐Vázquez, 2015; Méndez Zamora, Duran‐Meléndez, Aquino‐López, Santellano‐Estrada, & Silva‐Vázquez, 2016; Silva‐Vázquez, Duran‐Melendez, et al., 2017; Cázares‐Gallegos et al., 2019; Charles Avilés et al., 2019; Hernández‐Coronado et al., 2019; Sánchez‐Zamora et al., 2019; Herrera‐Balandrano et al., 2020). These oregano essential oils have gained attention due to antioxidant properties conferred by a mixture of phenolic monoterpenes including thymol, carvacrol, and their precursor p‐cymene (Silva‐Vázquez, García‐Macías, Duran‐Meléndez, Hume, & Méndez‐Zamora, 2017).

In the current study, Mexican oregano essential oils (MOEO) from *Lippia berlandieri* Schauer and *Poliomintha longiflora* Gray were compared to butylated hydroxytoluene as they affected preservation and shelf life of ground beef over 7 days of storage at 4°C, with evaluations of psychochemical traits, microbiology, antioxidant capacity, texture, and sensory properties.

## 2 MATERIAL AND METHODS

### 2.1 Experimental design

A randomized complete design of four treatments was used. The treatments were GB1 = GB control, GB2 = GB +100 mg/kg of Bht, GB3 = GB +100 mg/kg of Lb, and GB4 = GB +100 mg/kg of Pl. The treatments were evaluated at 1, 4, and 7 days of storage at 4°C, and each treatment was made in two replicates of 1.5 kg of GB each per storage day and a total of 9.0 kg of GB per treatment. The MOEO (Natural Solutions Company SMI, Jimenez, Chihuahua, Mexico) were obtained from oregano leaves of plants grown in Chihuahua, Mexico. The leaves were dried at room temperature, and essential oils were extracted using steam distillation. Oil composition was determined by gas chromatography (PerkinElmer Clarus 600 and SQ8 GC/MS; PerkinElmer Inc., Waltham, MA, USA) equipped with a flame ionization detector, and a Perkin Elmer PE‐1 capillary column (30 m × 0.25 mm, 0.25 μm film thickness) for separation of the oil components (Dunford and Silva Vazquez, 2005). The Lb MOEO contained 60.00% carvacrol and 3.91% thymol, while Pl MOEO presented 13.80% carvacrol and 28.40% thymol as principal compounds (Silva‐Vázquez et al., 2018). Treatment doses of 100 mg/kg were established according to General Standard for Food Additives CODEX STAN^192^‐, ^1995^ (revision 2019; 08.3 point, processed comminuted meat, poultry, and game products, considering the notes 15, 130, 162, XS88, XS89, and XS98) on Bht used in foods.

### 2.2 Meat preparation

Lean beef (biceps femoris) was purchased from a local market specializing in meat products and which ensured raw meat quality according to Mexican official standard NOM‐008‐ZOO (1994). The treatment preparations consisted of 36 kg, divided into two replicates of 1.5 kg per treatment for each day at 1, 4, and 7 days (two replicates of 1.5 kg/treatment/day). The meat, maintained at 4°C, was ground through a 3/8‐in (9.5‐mm) grind plate using a TORREY^®^ grinder (Model MV‐22R‐SS; Grupo Torrey, S.A. de C.V., Nuevo Leon, Mexico). Next, the GB was put onto plastic trays. For GB2, GB3, and GB4, 100 mg/kg of each MOEO and Bht, respectively, was added and mixed manually for 5 min. Then, the GB preparations were placed into a TORREY® mixer (Model MV‐25; Grupo Torrey, S.A. de C.V., Nuevo Leon, Mexico) for 10 min, mixing slowly. Finally, the GB was packed into Ziploc bags, coded per treatment replication and day, and stored at 4°C for evaluation at 1, 4, and 7 days.

### 2.3 Physicochemical analysis

Meat pH was measured with a puncture electrode (HI 99163, Hanna Instruments WoonSocket, RI, USA) at 4°C. Meat color values for variables of lightness (L*), redness (a*), yellowness (b*), Chroma (saturation index), and Hue angle were measured on the surfaces of the lean meat samples in areas without fat and connective tissue using a colorimeter (CR‐400 Konica Minolta®, Tokyo, Japan; Illuminant/Observer: D65/10) calibrated with a standard white plate and specified by CIE Lab System (CIE, 1976). Total color change (ΔE) and browning index (BI) were calculated according to equations used by Bozkurt and Bayram (2006), and the colorimeter calibration values $L_{0}^{*}$ = 94.18, $a_{0}^{*}$ = −0.43, and $b_{0}^{*}$ = 3.98 to estimate ΔE and BI. Water‐holding capacity (WHC) was determined using the compression method according to Tsai and Ockerman (1981) and Méndez‐Zamora et al. (2015) with slight modifications. A total of 0.3 g of meat was placed between two filter papers and placed between two 12 × 12 cm plexiglass plates, and a force of 4.0 kg was applied for 20 min; the initial (IW) and final weights (FW) were recorded, and WHC was estimated as WHC = 100‐[((IW‐FW)/IW × 100]. The physicochemical variables were determinate in six different subgroups per treatment/replicate (*n* = 12).

### 2.4 **C**ooking loss and meat texture

To determine cooking loss and meat texture, 50.92 ± 0.28 g of GB was placed into 50‐ml Eppendorf conical tubes (Eppendorf®, Hamburg, Germany; six tubes per replicate, *n* = 12) and cooked by immersion for 1 hr in water at 75.0 ± 0.1°C. Then, the samples were cooled at room temperature (20°C) for 45 min. The samples were removed from the tubes and carefully drained. Raw and cooked weights of GB were recorded to evaluate cooking loss percentage as ((raw weight ‐ cooked weight)/raw weight piece) × 100. Shear force measurements and texture profile analysis were carried out with a TA.XT Plus texturometer (Stable Micro Systems, Surrey, England). Shear force (g_f_) was measured using a Warner–Bratzler shear blade with a triangular slot cutting edge. Standardized cylinders (3.5 cm long and 2.0 cm in diameter) were used to evaluate shear force. Test conditions used in the instrument were velocities of 2 mm/s pretest, 2 mms/s during the test, 10 mm/s posttest, and a distance of 30 mm. The shear force value was taken from the maximum point of the curve generated. Texture profile analysis was determined using standardized cylinders (1.5 cm high and 2.0 cm in diameter). A cylindrical piston (75 mm in diameter) was used to compress the sample during two test cycles, compressing the sample up to 60% of the original height within a time span of 5 s between cycles. Force–time curves of deformation were obtained from the conditions established in the texturometer. The velocities used were 2.0 mm/s pretest, 5.0 mm/s during the test, and 5.0 mm/s posttest. The following parameters were recorded according to Bourne (1978): hardness (g), adhesiveness (g/s), springiness (mm), cohesiveness (dimensionless), gumminess (g), chewiness (g mm), and resilience (dimensionless).

### 2.5 **Antioxidant capacity and microbial analyse**s

Radical 2,2‐diphenyl‐1‐picrylhydrazyl (DPPH) activity was determined according to method of Mielnik, Aaby, and Skrede (2003) with slight modifications. Samples of fresh GB were diluted 1:20 in ethanol (GB:ethanol). Fifty microliters of each diluted GB sample was added to 1 ml of DPPH in ethanol solution (25 mg/L). Reaction mixtures were incubated at 25°C for 20 min in darkness. Samples optical densities were measured in a spectrophotometer (Shimadzu UV‐VIS 1,800, Kyoto, Japan) at 517 nm. Spectrophotometer readings were carried out in triplicate for each of two replicates per treatment (*n* = 6 per treatment).

Bacterial colony counts were carried out according to NOM‐110‐SSA1 (1994) and NOM‐092‐SSA1 (1994) in triplicate for each of two replicates at 1, 4, and 7 days per replicate (*n* = 6 per treatment per day). A total of 10 g per sample were collected aseptically, transferred to sterile polyethylene bags to which was added 90 ml of sterile phosphate buffer (pH 6.5). Each sample was subjected to three 1.5‐min mixing cycles in a Stomacher (Seward Laboratory, London, UK). A 1‐mL portion was transferred onto a nutrient agar plate (Laboratorios CONDA S.A., Madrid, Spain). Total aerobic mesophilic and psychrophilic bacteria colony counts were determined on plates incubated for 48 hr at 30°C and 4°C, respectively. The lactic acid bacteria (LAB) colony counts were carried out on DeMan, Rogosa, and Sharpe agar (MRS; Laboratorios CONDA S.A., Madrid, Spain), and the plates were incubated at 37°C for 72 hr. Microbial colony‐forming unit counts were transformed to log_10_ CFU/g of sample for comparisons.

### 2.6 Sensory evaluation

Sensory attributes were measured according to procedures of Anzaldúa‐Morales (1994) and Meilgaard, Civille, and Carr (2006) to determine the satisfaction levels of 30 consumers offered GB raw stored for 7 days at 4°C. Each semitrained consumer (women and men 20–30 years old) randomly evaluated 10.0 g of raw GB at 4°C and placed into plastic cups encoded with three numbers chosen at random. The sensorial test was performed in the Sensory Laboratory outfitted with individual booths with a sink, table, and chair in each. The attributes evaluated were red color, odor, brightness, and firmness, and overall acceptability recorded in a questionnaire, considering a 7‐point hedonic scale (7 = liked very much and 1 = disliked very much), where the participants indicated the sensory preference per attribute. The experimental protocol numbered 014/17 was approved by the Postgraduate and Research Subdirectorate, Facultad de Agronomia, Universidad Autonoma de Nuevo Leon.

### 2.7 Statistical analysis

An analysis of variance was performed using the proc GLM of SAS (2006) and the next model statistical: y_ijk_ = µ + Ƭ_i_ + δ_j_ + (Ƭδ)_ij_ + Ԑ_ijk_, where y_ijk_ = physicochemical, textural, microbiological, antioxidant, and sensory variables evaluated over time; µ = general media; Ƭ_i_ = fixed effect of *i*‐th treatment (GB1‐control, GB2‐Bht, GB3‐Lb, and GB4‐Pl); δ_j_ = effect of *j*‐th evaluation day (1, 4, and 7 days); (Ƭδ)_ij_ = effect of the interaction between the *i*‐th treatment and the *j*‐th day; and Ԑ*_ijk_* = random error normally independently distributed with media of zero and variance σ^2^ [Ԑ*_ijk_* ~ NID (0, σ^2^)]. The statistical model of sensorial analysis considered a complete random block design, where each consumer was the block effect (β_j_). A significance level of *p* < .05 was used to assess significant differences between treatment means, days, and interaction. When the fixed effects and its interaction had significant effect, the means were compared using Adjust = Tukey (SAS, 2006).

## 3 RESULTS AND DISCUSSION

### 3.1 Physicochemical analysis

The pH and color parameters are traits evaluated in raw meat as predictors of quality and physicochemical characteristics (Warriss, 2000). The pH values at Day 1 (Table 1) were similar to those reported by Yin, Xing, Zhou, and Zhang (2016), who evaluated the effects of rosemary extracts (53, 33, and 55 g/kg) on raw ground pork patties, but those authors found that sample pH decreased steadily to the last day of evaluation at 10 days, which contrasted with the current study in which the pH increased slightly. Corral *et al*. (2013) indicated that the decreasing meat pH could be due to lactic acid bacteria synthetizing lactic acid. Hence, pH behavior at Day 7 in the current study may indicate a possible inactivation of the lactic acid bacteria due to influences of the oregano oil phenolic components. Color responses showed significant differences (*p* < .05) for b* and Hue angle of GB in the treatments and days interaction ((Ƭδ)*_ij_*; Table 1), and a* did not show significant difference (*p* > .05) in (Ƭδ)*_ij_*. Treatments (Ƭ*_i_*) were different (*p* < .05) for L* and a*, while the days were different (*p* < .05) for pH and WHC. Values of L* were higher (*p* < .05) at days 1, 4, and 7 for GB3 compared to GB1. Ismail, Lee, Ko, and Ahn (2008) obtained similar results when using natural antioxidants (0.05% ascorbic acid, 0.01% α‐tocopherol and 0.01% sesamol) on irradiated ground beef. Sample a* was higher (*p* < .05) for GB1 at Day 1 and lower for GB3 at Day 4, but GB2 (Bht) and GB4 (Pl) maintained level (*p* < .05) a* values, showing the best treatments to conserve a*. These results are improvements over those obtained with the rosemary extract (3,000 ppm) as examined by Balentine et al. (2006) and grape seed extract (0 and 250 mg GSE/kg of product) as studied by Gómez et al. (2016) in ground beef and hence suggest that MOEO may better preserve physicochemical properties than rosemary extract. It is thought that MOEO decreases myoglobin degradation, in which autoxidation is controlled by natural antioxidants to reduce the rate of myoglobin color degradation (Balentine et al., 2006). For b*, GB2 (Bht) presented the highest (*p* < .05) value at Day 3 and GB1 the lowest (*p* < .05) value, while GB2 and GB3 (Lb) were the best treatments to conserve color. These results were contrary to those of Belantine et al. (2006) in which responses were comparatively low for color variables. Chroma and Hue were different (*p* < .05) for fixed effects and interaction. Chroma was higher (*p* < .05) for GB2 and GB4 at days 4 and 7, but GB3 was lower (*p* < .05) at Day 4 and GB1 at Day 7. Hue angle was higher (*p* < .05) for GB3 at days 1, 4, and 7, while GB1 presented lower (*p* < .05) values. These results were similar to those reported by Balentine et al. (2006) over the first hours of the experiment; however, adequate color values were not maintained by rosemary extract; in contrast, the MOEO maintained the color parameters over 7 days. The control group GB1 had the highest (*p* < .05) value for ΔE and BI at Day 1, and GB3 (Lb) was the lowest (*p* < .05) for ΔE at days 4 and 7, while GB1 was the lowest (*p* < .05) at Day 7 for BI. Again, these results suggest that MOEO may substitute as a preservative for GB. Table 2 shows pH and cooking loss for cooked GB. The pH was affected (*p* < .05) at days and treatment interactions, while cooking loss was influenced (*p* < .05) by treatments. The pH at Day 7 was higher (*p* < .05) for GB1 and lower (*p* < .05) for GB3 and GB4. These responses possibly reflect influences of MOEO pH (4.60) on this parameter. The cooking loss showed higher (*p* < .05) values at each day evaluated, and GB1 obtaining the lowest values at Day 1 and Day 7, and GB3 was lower at Day 4. These data obtained for pH and cooking loss demonstrated that MOEO decreased meat degradation (proteolysis and lipolysis) during storage for 7 days and 4°C; hence, protein and fat preserved their native chemical structure and when MOEO‐treated meat was cooked, the properties of these components were not affected by thermic treatment.

**Table 1:** Physicochemical analysis over 7 days of storage at 4°C of raw beef ground treated with Mexican oregano essential oils and butylated hydroxytoluene

Days/ Treatments^†^	Variables^‡^								
	pH	WHC (%)	L*	a*	b*	Chroma	Hue	ΔE	BI
Day 1									
GB1	5.88	60.58	49.31^c^	27.18^a;A^	15.33^a;A^	31.30ª^;A^	29.45^a;AB^	53.95^a;A^	75.03^a;A^
GB2	5.92	61.01	50.84^b^	26.39^a;A^	15.44^a;A^	30.59ª^;AB^	30.37^a;AB^	52.30^a;AB^	71.92^a;AB^
GB3	5.86	61.30	52.66^a^	25.50^a;A^	15.85^a;A^	30.01ª^;ABC^	31.82^a;AB^	50.43^c;BC^	69.34^ab;ABC^
GB4	5.92	61.40	51.84^a^	25.72^a;A^	15.26^a;A^	29.99ª^;ABC^	30.80^a;AB^	51.08^b;BC^	69.08^b;ABC^
Day 4									
GB1	5.99	60.00	49.49^b^	20.24^a;B^	13.65^a;AB^	24.43^b;E^	34.13^a;A^	50.20^a;BC^	60.87^ab;CDE^
GB2	6.00	60.62	50.14^a^	22.96^a;B^	14.28^a;AB^	27.03ª^;BCDE^	32.36^b;AB^	51.03^a;BC^	65.29^a;BCDE^
GB3	6.01	60.90	51.27^a^	20.02^a;B^	13.99^a;AB^	22.16^c;E^	35.49^a;A^	48.63^a;C^	59.17^b;DE^
GB4	6.03	61.47	49.84^a^	23.18^a;B^	14.24^a;AB^	27.25ª^;BCDE^	32.32^c;AB^	51.45^a;B^	65.77^a;BCDE^
Day 7									
GB1	6.03	66.37	47.81^b^	22.41^a;B^	11.00^b;B^	24.99^c;DE^	26.21^a;B^	52.19^ab;AB^	58.47^c;E^
GB2	6.05	63.86	49.60^a^	25.50^a;AB^	13.71^a;AB^	29.04ª^;ABC^	28.26^a;B^	52.57^a;AB^	67.73^a;ABCD^
GB3	6.00	65.40	50.48^a^	23.47^a;AB^	12.70^a;AB^	26.40ª^;CDE^	28.46^a;B^	50.60^b;BC^	61.15^b;CDE^
GB4	5.90	65.21	49.76^a^	24.09^a;AB^	12.44^a;B^	28.64ª^;ABCD^	27.45^a;B^	51.49^a;AB^	62.15^a;CDE^
*SEM*	0.06	0.99	0.38	0.81	0.32	0.97	0.59	0.52	1.76
P‐value									
Treatments (Ƭ*_i_*)	0.8742	0.7312	< 0.0001	0.0117	0.0002	0.0015	0.0003	0.0001	0.0080
Days (δ*_j_*)	0.0251	< 0.0001	< 0.0001	< 0.0001	< 0.0001	< 0.0001	< 0.0001	0.0001	0.0001
Interaction (Ƭδ)*_ij_*	0.6979	0.7375	0.1054	0.0679	0.0034	0.0264	0.0149	0.0110	0.0090

^a‐d^Means (*n* = 12/treatment/day) within the same column and within each treatment and at each day with different lowercase superscripts differ significantly when the *p*‐value of (Ƭ*_j_*) < .05.

^A‐E^Means (*n* = 12/treatment/day) within the same column, for all treatments and for all days, with different uppercase superscripts differ significantly when the *p*‐value of (Ƭδ)*_ij_* < .05.

^†^ GB1 = ground beef (GB; control); GB2 = GB +100 mg/kg of butylated hydroxytoluene; GB3 = GB +100 mg/kg of *Lippia berlandieri* Schauer essential oil; and GB4 = GB +100 mg/kg of *Poliomintha longiflora* Gray essential oil. *SEM* = standard error of means. Ƭ*_i_* = fixed effect of *i*‐th treatment (GB1, GB2, GB3, and GB4); δ*_j_* = effect of *j*‐th evaluation day (1, 4, and 7 days); (Ƭδ)*_ij_* = effect of the interaction between the *i*‐th treatment and the *j*‐th day.

^‡^ WHC = water‐holding capacity; L* = lightness; a* = redness; b* = yellowness; Chroma = saturation index; Hue = Hue angle (tonality); ΔE = total color change; BI = browning index.

**Table 2:** Sample pH and cooking loss over 7 days of storage at 4°C of ground beef treated with Mexican oregano essential oils and butylated hydroxytoluene

Days/ Treatments^†^	pH	Cooking loss (%)
Day 1		
GB1	6.24^a;DEF^	14.61^b^
GB2	6.20^a;EF^	19.16^a^
GB3	6.18^a;F^	15.31^b^
GB4	6.20^a;EF^	15.29^b^
Day 4		
GB1	6.29^a;CDEF^	17.43^b^
GB2	6.34^a;BCDE^	19.82^a^
GB3	6.31^a;BCDEF^	16.93^b^
GB4	6.32^a;BCDE^	17.25^b^
Day 7		
GB1	6.60^a;A^	19.02^b^
GB2	6.44^b;B^	21.95^a^
GB3	6.38^b;BC^	19.89^ab^
GB4	6.35^b;BCD^	20.99^ab^
*SEM*	0.03	0.60
P‐value		
Treatments (Ƭ*_i_*)	0.0022	< 0.0001
Days (δ*_j_*)	< 0.0001	< 0.0001
Interaction (Ƭδ)*_ij_*	0.0003	0.2087

^a‐d^Means (*n* = 12/treatment/day) within the same column and within each treatment and at each day with different lowercase superscripts differ significantly when the *p*‐value of (Ƭ*_j_*) < .05.

^A‐C^Means (*n* = 12/treatment/day) within the same column, for all treatments and for all days, with different uppercase superscripts differ significantly when the *p*‐value of (Ƭδ)*_ij_* < .05.

^†^ GB1 = ground beef (GB; control); GB2 = GB +100 mg/kg of butylated hydroxytoluene; GB3 = GB +100 mg/kg of *Lippia berlandieri* Schauer essential oil; and GB4 = GB +100 mg/kg of *Poliomintha longiflora* Gray essential oil. *SEM* = standard error of means. Ƭ*_i_* = fixed effect of *i*‐th treatment (GB1, GB2, GB3, and GB4); δ*_j_* = effect of *j*‐th evaluation day (1, 4, and 7 days); (Ƭδ)*_ij_* = effect of the interaction between the *i*‐th treatment and the *j*‐th day.

### 3.2 Antioxidant capacity and microbial analyses

In the current study, the effect of days and treatment interaction was significant (*p* < .05) for total antioxidant capacity (AC) (Table 3). This result indicated that treatment and days had effects on that variable. In fact, AC for GB2 (Bht) and GB4 (Pl) were lower (*p* < .05) and GB1 (control) was higher (*p* < .05) at Day 1, while GB3 (Lb) was the highest (*p* < .05) at Day 7, and followed by GB1, GB2, and GB4. Those differences could be due to the different affinities of free radicals to scavenge the various antioxidant groups present in different meat samples (Serpen, Gökmen, & Fogliano, 2012). In the current study, these differences could be due to the free radical affinities of phenolic carvacrol and thymol in MOEO and the ripening and preservative processes over 7 days. The AC of phenolic compounds is determined by their quantity and chemical structures (Kim et al., 2013), and the AC describes the capacity of muscle to resist oxidation processes (Serpen et al., 2012). The Mexican oregano oils contain high concentrations of carvacrol (Lb) and thymol (Pl) (Burt, 2004), hence benefiting antioxidant activity in GB by inhibiting lipid oxidation (Kim et al., 2013) and increasing oxidative stability (Mohamed et al., 2011).

**Table 3:** Antioxidant capacity and microbial analyses over 7 days of storage at 4°C of raw ground beef treated with Mexican oregano essential oils and butylated hydroxytoluene

Days/Treatments^†^	Antioxidant capacity (%)	Bacteria counts (log CFU/g)		
	DPPH	Mesophilic	Psychrophilic	LAB
Day 1				
GB1	4.95^a;B^	6.13^a;C^	4.06^F^	4.96^a;D^
GB2	1.99^b;C^	5.98^ab;CD^	3.76^F^	4.45^b;E^
GB3	0.97^c;CD^	5.75^b;D^	4.22^EF^	4.28^b;E^
GB4	0.00^c;D^	5.74^b;D^	4.18^EF^	4.20^b;E^
Day 4				
GB1	6.07^a;B^	7.16^a;B^	4.95^BCD^	5.54ª^;BC^
GB2	2.09^b;C^	7.18^a;B^	5.05^ABCD^	5.65ª^;B^
GB3	2.04^b;C^	7.02^a;B^	4.90^CD^	5.26^b;CD^
GB4	1.22^b;CD^	6.94^a;B^	4.71^DE^	5.04^b;D^
Day 7				
GB1	5.35^b;B^	7.89^a;A^	5.55^A^	5.67^b;B^
GB2	5.20^b;B^	7.98^a;A^	5.55^A^	5.74^b;B^
GB3	10.51^a;A^	8.04^a;A^	5.45^AB^	6.22^a;A^
GB4	0.56^c;CD^	7.98^a;A^	5.44^ABC^	5.87^ab;AB^
*SEM*	0.34	0.07	0.11	0.08
P‐value				
Treatments (Ƭ*_i_*)	< 0.0001	0.0194	0.7437	< 0.0001
Days (δ*_j_*)	< 0.0001	< 0.0001	< 0.0001	< 0.0001
Interaction (Ƭδ)*_ij_*	< 0.0001	0.0220	0.0397	< 0.0001

^a‐d^Means (*n* = 6/treatment/day) within the same column and within each treatment and at each day with different lowercase superscripts differ significantly when the *p*‐value of (Ƭ*_j_*) < .05.

^A‐F^Means (*n* = 6/treatment/day) within the same column, for all treatments and for all days, with different uppercase superscripts differ significantly when the *p*‐value of (Ƭδ)*_ij_* < .05.

^†^ GB1 = ground beef (GB; control); GB2 = GB +100 mg/kg of butylated hydroxytoluene; GB3 = GB +100 mg/kg of *Lippia berlandieri* Schauer essential oil; and GB4 = GB +100 mg/kg of *Poliomintha longiflora* Gray essential oil. *SEM* = standard error of means. Ƭ*_i_* = fixed effect of *i*‐th treatment (GB1, GB2, GB3, and GB4); δ*_j_* = effect of *j*‐th evaluation day (1, 4, and 7 days); (Ƭδ)*_ij_* = effect of the interaction between the *i*‐th treatment and the *j*‐th day. CFU = colony‐forming units; DPPH = 2,2‐diphenyl‐1‐picrylhydrazyl; LAB = lactic acid bacteria.

Oregano essential oils contain compounds that interfere with the microbial growth and proliferation and hence could reduce meat spoilage. In the current study, statistical differences for microbial colony counts were obtained for treatments with MOEO (Table 3). Colony counts for mesophilic, psychrophilic, and LAB were different (*p* < .05) at days and treatment interaction. These variables increased (*p* < .05) over time, Day 7 > Day 1. Mesophilic counts were higher (*p* < .05) for GB1 at Day 1, but lower (*p* < .05) for GB3 and GB4 at these days. Essential oils containing carvacrol and thymol showed strong antibacterial activities (Mith et al., 2014). These activities could be attributed to GB3 and GB4 treatments, because they contain high carvacrol and thymol, respectively. At day 4 and Day 7, all treatments presented similar (*p* > .05) values, with GB3 at Day 4 colony counts being slightly higher (*p* > .05) than the other treatments. Najjaa et al. (2020) obtained similar results for aerobic mesophilic flora enumeration in ground meat at 6 days using microcapsule *Allium sativum* (garlic) extracts (5, 10, 15, and 20%). Those authors indicated that garlic could extend the shelf life of minced meat and maintained satisfactory and acceptable qualities. Regarding psychrophilic counts, GB1 showed the highest (*p* < .05) values at Day 1, while all treatments exhibited similar colony counts (*p* > .05) Day 4 and Day 7. Dhifi et al. (2020) found effect of *Myrtus communis* (common myrtle plant) flower essential oils (0.4 and 0.8% v/w combined with nisin at 500 AU/g) on psychrophilic counts (low values) in raw minced beef stored at 7 days at 4°C, indicating that these results may be attributed to antioxidant and antimicrobial activities of essential oils against foodborne pathogens due to its phenolic compounds. Colony counts for LAB were highest (*p* < .05) for GB1 at Day 1, highest (*p* < .05) for GB1 and GB2 and lowest (*p* < .05) for these two treatments at Day 7. Counts were higher (*p* < .05) for GB3 at Day 7. Yin et al. (2016) evaluated rosemary extract on ground pork for hamburgers and suggested that the essential oil active compounds modified bacterial cell membranes and its permeability, inhibiting bacterial proliferation. Oregano essential oil active compounds carvacrol and thymol similarly inhibit bacterial activity and proliferation (Burt, 2004).

### 3.3 Texture analysis

Table 4 presents the texture analysis over 7 days of storage of ground beef treated with Mexican oregano oils and butylated hydroxytoluene (Bht). Hardness, springiness, cohesiveness, and resilience were different (*p* < .05) between treatments and days, while shear force, gumminess, and chewiness were different (*p* < .05) for treatments. Adhesiveness did not present (*p* > .05) differences between treatments and interaction with days. Sample shear force was lower (*p* < .05) for GB1 at Day 1 and Day 4, and shear force for all treatments was similar (*p* > .05) at Day 7, although at Day 7 shear force for treatments GB2 to GB4 was numerically (*p* > .05) higher than GB1 shear force. Gumminess had was highest (*p* < .05) for GB4 at 4 days and GB1 the lowest (*p* < .05). Hardness was similar (*p* > .05) for all treatments at Day 1, and lowest (*p* < .05) for GB3 (Lb) and GB1 (control) at Day 1 and Day 7, respectively. Reihani, Thuan‐Chew, Huda, and Easa (2014) indicated that the antioxidant compounds have a protective effect in the muscle membrane against lipid oxidation, helping to maintain membrane integrity of muscle fibers and decrease moisture loss (Lara, Gutierrez, Timón, & Andrés, 2011; Maqsood, Benjakul, & Balange, 2012). Hence, hardness, springiness, cohesiveness, shear force, gumminess, chewiness, and resilience could be improved by the antioxidant capacities of compounds added to the samples, which conserved the meat structure during storage over 7 days. In the current study, essential oils components are antioxidant compounds, which, due to their aromatic chemical structures, bond with free radicals, thus improving the proportion of collagen types I and III, which after thermic treatment during cooking of the meat tenderness was not affected (Monteschio et al., 2019). Springiness and cohesiveness for treatment GB3 (Lb) at Day 1 had the lowest (*p* < .05) values, while GB4 (Pl) for springiness and GB1 (control) for cohesiveness were lowest (*p* < .05) at Day 4. Springiness values for GB3 were lowest (*p* < .05) at Day 7, and all treatments at Day 7 had similar (*p* > .05) values for cohesiveness. Reihani et al. (2014) reported springiness values lower than those from the current study. There were no differences (*p* > .05) in gumminess and chewiness, respectively, in comparisons for all three days taken together. Values for gumminess were not different (*p* > .05) at Day 1 and Day 7, respectively, while at Day 4 gumminess values for GB3 were lowest (*p* < .05). Similarly, values for chewiness were not different (*p* > .05) at Day 1 and Day 7, respectively, although GB4 had numerically higher (*p* > .05) values than the other groups over all days. At Day 4, GB1 values for chewiness were lower (*p* < .05) than those for the other treatments. Treatment GB3 was lowest (*p* < .05) for resilience at Day 1, all treatments were similar to each other at Day 4 and Day 7. Although there was overlap in differences for comparisons of all treatments over the three days, GB1 and GB3 presented the lowest (*p* < .05) values at Day 1, while GB1 and GB2 at Day 7 presented the highest (*p* < .05) values. These results can indicate that type III collagen in association with perimysium fibers and the endomysium sheath benefit meat texture properties (Monteschio et al., 2019).

**Table 4:** Texture analysis over 7 days of storage at 4°C of ground beef treated with Mexican oregano essential oils and butylated hydroxytoluene

Days/ Treatments^†^	Shear force (g_f_)^‡^	Hardness (g)	Adhesiveness (g/s)	Springiness (mm)	Cohesiveness	Gumminess (g)	Chewiness (g mm)	Resilience
Day 1								
GB1	920.27^b^	6,416.67^a;AB^	−2.40	0.81^a;AB^	0.39^a;AB^	2,491.08^a^	2032.00^a^	0.14^a;B^
GB2	1,199.03^a^	5,969.43^a;AB^	1.99	0.80^a;AB^	0.43^a;AB^	2,576.11^a^	2068.15^a^	0.16^a;AB^
GB3	1,046.00^a^	6,724.61^a;AB^	−0.55	0.77^b;B^	0.37^b;B^	2,521.35^a^	1943.00^a^	0.13^b;B^
GB4	1,129.37^a^	6,978.99^a;AB^	−0.70	0.81^a;AB^	0.43^a;AB^	2,987.79^a^	2,416.86^a^	0.16^a;AB^
Day 4								
GB1	1,026.77^b^	5,993.74^c;B^	−0.40	0.86^a;A^	0.37^b;B^	2,184.16^c^	1877.23^b^	0.15^a;AB^
GB2	1,295.07^a^	7,109.88^a;A^	−1.97	0.84^a;A^	0.42^a;AB^	2,971.22^a^	2,486.06^a^	0.16^a;AB^
GB3	1,166.29^a^	6,358.86^b;AB^	−2.82	0.83^a;AB^	0.39^a;AB^	2,518.55^b^	2099.15^a^	0.16^a;AB^
GB4	1,298.40^a^	7,685.21^a;A^	−0.38	0.82^b;AB^	0.41^a;AB^	3,160.55^a^	2,597.93^a^	0.16^a;AB^
Day 7								
GB1	1,381.26^a^	5,047.48^b;B^	−10.99	0.86^a;A^	0.46^a;AB^	2,330.97^a^	1996.63^a^	0.18^a;AB^
GB2	1,448.87^a^	5,454.41^a;AB^	−6.17	0.84^a;A^	0.48^a;A^	2,613.98^a^	2,199.45^a^	0.19^a;A^
GB3	1,404.45^a^	5,664.00^a;AB^	−2.62	0.81^b;AB^	0.44^a;AB^	2,497.66^a^	2038.69^a^	0.18^a;AB^
GB4	1,445.91^a^	6,553.57^a;AB^	−0.29	0.83^a;AB^	0.45^a;AB^	2,930.81^a^	2,436.40^a^	0.17^a;AB^
*SEM*	54.51	260.20	2.29	0.01	0.01	135.59	111.84	0.01
P‐value								
Treatments (Ƭ*_i_*)	< 0.0001	< 0.0001	0.1976	< 0.0001	< 0.0001	< 0.0001	< 0.0001	0.0064
Days (δ*_j_*)	< 0.0001	< 0.0001	0.0263	< 0.0001	< 0.0001	0.4581	0.1543	< 0.0001
Interaction (Ƭδ)*_ij_*	0.4144	0.0342	0.2599	0.0372	0.0403	0.2756	0.3288	0.0107

^a‐d^Means (*n* = 12/treatment/day) within the same column and within each treatment and at each day with different lowercase superscripts differ significantly when the *p*‐value of (Ƭ*_j_*) < .05.

^A‐C^Means (*n* = 12/treatment/day) within the same column, for all treatments and for all days, with different uppercase superscripts differ significantly when the *p*‐value of (Ƭδ)*_ij_* < .05.

^†^ GB1 = ground beef (GB; control); GB2 = GB +100 mg/kg of butylated hydroxytoluene; GB3 = GB +100 mg/kg of *Lippia berlandieri* Schauer essential oil; and GB4 = GB +100 mg/kg of *Poliomintha longiflora* Gray essential oil. *SEM* = standard error of means. Ƭ*_i_* = fixed effect of *i*‐th treatment (GB1, GB2, GB3, and GB4); δ*_j_* = effect of *j*‐th evaluation day (1, 4, and 7 days); (Ƭδ)*_ij_* = effect of the interaction between the *i*‐th treatment and the *j*‐th day.

### 3.4 Sensory evaluation

Food acceptability by consumers establishes future market direction and influences applications of new technologies in the food processing industry (Mohamed et al., 2011). In the current study, the consumers did not express differences (*p* > .05) in the sensory evaluation of treated (Table 5) raw ground beef. In summary, red color, brightness, odor, firmness, and overall acceptability were greater than 4.30 but less than 5.27 points (liked moderately) on the 7‐point hedonic scale. These results showed that MOEO did not influence acceptance of sensory attributes for treatments with Lb (GB3) and Pl (GB4). In contrast, Ghabraie, Vu, Tata, Salmieri, and Lacroix (2016) found differences in sensorial evaluation of ground beef treated with combined Chinese cinnamon and cinnamon bark essential oils (0.0125, 0.025, 0.05, 0.1, and 0.2%), with the lowest acceptance for smell and taste given to high concentrations of the oils. The addition of 0.1% of garlic and 0.5% of onion in irradiated raw ground beef increased onion/garlic aroma and ground beef color, because the additives contain greater amounts of sulfur compounds and are more efficient in masking irradiation aroma (Yang et al., 2011). Additionally, 0.04% of rosemary extract in irradiated ground beef decreased odor, color, and acceptability of meat stored at refrigeration temperature (5°C) for 40 days (Mohamed et al., 2011).

**Table 5:** Sensory evaluation of raw ground beef following 7 days of storage at 4°C and treated with Mexican oregano essential oils and butylated hydroxytoluene

Days/ Treatments^a^	Affective attributes^b^				
	Red color	Odor	Brightness	Firmness	Overall acceptability
GB1	5.20	4.69	5.00	5.27	5.13
GB2	5.13	4.79	4.83	5.07	5.07
GB3	4.86	4.80	4.60	4.93	5.07
GB4	5.13	4.33	4.57	4.93	5.00
*SEM*	0.22	0.24	0.21	0.21	0.21
P‐value	0.7162	0.5620	0.4132	0.6300	0.9784

^a^ GB1 = ground beef (GB; control); GB2 = GB +100 mg/kg of butylated hydroxytoluene; GB3 = GB +100 mg/kg of *Lippia berlandieri* Schauer essential oil; and GB4 = GB +100 mg/kg of *Poliomintha longiflora* Gray essential oil. *SEM* = standard error of means.

^b^ Means values (*n* = 30 consumers) within the same column were not different (*p* > .05).

## 4 CONCLUSIONS

Ground beef treated with *Lippia berlan*dieri Schauer and *Poliomintha longiflora* Gray essential oils (100 mg/kg) improved the color of ground beef over storage time. The two Mexican oregano essential oils improved numbers of lactic acid bacteria, increased meat hardness, shear force, and chewiness. These oregano oils did increase meat shelf life, which offers a potential option for the food industry to use natural ingredients in place of synthetic compounds to preserve ground beef quality and retain consumer acceptance. In conclusion, the oregano essential oils may provide extended shelf life for packaged meat products treated with these natural additives.

## 5 ETHICAL REVIEW

This research was approved by the Postgraduate and Research Subdirectorate‐Facultad de Agronomia, Universidad Autonoma de Nuevo Leon.

## **CONFLICT** **OF INTEREST**

The authors declare no conflict of interest.
